# Stitcher: A Surface Reconstruction Tool for Highly Gyrified Brains

**DOI:** 10.1007/s12021-024-09678-2

**Published:** 2024-10-10

**Authors:** Heitor Mynssen, Kamilla Avelino-de-Souza, Khallil Chaim, Vanessa Lanes Ribeiro, Nina Patzke, Bruno Mota

**Affiliations:** 1 Instituto de Ciências Biomédicas, Universidade Federal do Rio de Janeiro, Rio de Janeiro, 21941-971 Brazil; 2 Laboratório de Biologia Teórica e Matemática Experimental (MetaBIO), Instituto de Física, Universidade Federal do Rio de Janeiro, Rio de Janeiro, 21941-909 Brazil; 3 Rede Brasileira de Neurobiodiversidade, Instituto de Física, Universidade Federal do Rio de Janeiro, Rio de Janeiro, 21941-909 Brazil; 4 LIM44, Hospital das Clínicas HCFMUSP, Faculdade de Medicina, Universidade de São Paulo, São Paulo, SP 05403-010 Brazil; 5 Instituto Biopesca, Praia Grande, SP 11700-570 Brazil; 6 Faculty of Medicine, IMBB, HMU Health and Medical University, Potsdam, 14471 Germany; 7 Department of Biological Science, Faculty of Sciences, Hokkaido University, Sapporo, 060-0810 Japan

**Keywords:** Surface reconstruction, Cortical reconstruction, Cetaceans, Magnetic resonance imaging

## Abstract

Brain reconstruction, specially of the cerebral cortex, is a challenging task and even more so when it comes to highly gyrified brained animals. Here, we present Stitcher, a novel tool capable of generating such surfaces utilizing MRI data and manual segmentation. Stitcher makes a triangulation between consecutive brain slice segmentations by recursively adding edges that minimize the total length and simultaneously avoid self-intersection. We applied this new method to build the cortical surfaces of two dolphins: Guiana dolphin (*Sotalia guianensis*), Franciscana dolphin (*Pontoporia blainvillei*); and one pinniped: Steller sea lion (*Eumetopias jubatus*). Specifically in the case of *P. blainvillei*, two reconstructions at two different resolutions were made. Additionally, we also performed reconstructions for sub and non-cortical structures of Guiana dolphin. All our cortical mesh results show remarkable resemblance with the real anatomy of the brains, except *P. blainvillei* with low-resolution data. Sub and non-cortical meshes were also properly reconstructed and the spatial positioning of structures was preserved with respect to *S. guianensis* cerebral cortex. In a comparative perspective between methods, Stitcher presents compatible results for volumetric measurements when contrasted with other anatomical standard tools. In this way, Stitcher seems to be a viable pipeline for new neuroanatomical analysis, enhancing visualization and descriptions of non-primates species, and broadening the scope of compared neuroanatomy.

## Introduction

The cerebral cortex is a highly complex structure that integrates several overlapping organizational levels. Its complete description would need to incorporate cellular, biochemical, physiological, functional and structural information, and their complicated interactions and dependencies (Hansen et al., 2023; Wang et al., 2023; Pronold et al., 2023). Ultimately, all of these aspects must be spatially located.

A purely morphological ground truth, to which all other information can be mapped and co-registered, is a necessary foundation for integrative approaches to cortical structure and function. The most readily accessible morphological framework, obtained through Magnetic Resonance Imaging (MRI), is volumetric: Assigning different voxels to structures, such as White Matter (WM) and Gray Matter (GM) unambiguously partitions a brain into its component substructures. However, this Cartesian description is not a natural map for a thin sheet of GM wrapped around WM in complex gyrifications. For that, one needs to talk about surfaces: To describe cortical morphology in terms of the nested surfaces that delineate the boundaries of WM, GM and any other substructure of interest. But translating between voxel-based and surface-based descriptions is not always easy (Mota & Herculano-Houzel, 2015; Osechinskiy & Kruggel, 2012).

3D mesh models represent surfaces as connected sets of vertices, edges and (typically triangular) faces that define the boundaries of polyhedra, and are presumed to be good approximations for the underlying smooth physical surfaces (Arndt et al., 1994; Tisserand et al., 2002; Navarrete et al., 2018). They are usually rendered at a much higher resolution than the voxel-based segmentation they are derived from, to allow for a smooth interpolation of the boundaries between different structures. This description lends itself well to measurements of surface area and gyrification indexes, and form the basis for a range of morphometric brain analyses (Mota & Herculano-Houzel, 2015; Wang et al., 2023). Such area-based analyzes are much better at showing how intricately the different part of a brain fit together.

MRI segmentation provides the data required to build both cortical and subcortical surface meshes. Images generated from MRI acquisition can be fed directly into automatic segmentation and reconstruction tools, which will generate the boundary meshes between the segmented sub-structures. For example, FreeSurfer (FS; Fischl, 2012) is a fully automated software that aims to process MRI brain data without the need for human supervision. FS includes routines capable of stripping the skull and other tissues surrounding the brain, classify brain voxels (*i.e.* GM, WM and CSF) and then generate high-resolution meshes smoothly delineating the boundaries between these structures.

However, FS has limitations that can sometimes complicate or hinder surface reconstruction, depending on the tissue of interest (Collins & Evans, 1997; Cherbuin et al., 2009). Notably, its segmentation algorithm is guided by previously segmented data sets, which are used to create image masks for its voxel labeling algorithms (Dale et al., 1999). This prevents the usage of FS for cetaceans and many other mammals with less well known brain anatomy, for which datasets may comprise from one to at best a handful of specimens. Given these challenges, a fully automated segmentation of non-model brains is still beyond reach, and manual techniques, supported by expert knowledge, still play a crucial role.

On the other end of the automation spectrum, manual techniques rely on the delineation of brain structures based on anatomical landmarks provided by atlases. Unlike automatic methods, they require anatomical expertise to identify and label the region of interest. There are two primary methods for manually segmenting brain structures from MRI: stereology with point counting (García-Fiñana et al., 2003, 2009), and tracing methods (Keller & Roberts, 2010). As our main contribution in the present study, we employed the latter, using a drawing tablet to trace contours of interest on evenly spaced selected MR slices of two cetacean and one piniped species: Guiana dolphin (*Sotalia guianeneis*), Franciscana dolphin (*Pontoporia blainvillei*) and Steller sea lion (*Eumetopias jubatus*). The traced contours on adjacent slices are then automatically stitched together to generate the surfaces that interpolate between contours.

Like any technique, manual methods have pros and cons. They are known to be more accurate and reliable when performed by skilled anatomists. However, they can be time-consuming, prone to bias, and impractical for large datasets. Prospectively, much of this work may in the future be automatized through machine learning models; but these cannot entirely replace human expertise (Topol, 2019). Instead, machine learning and human expertise are likely to complement each other, improving efficiency and consistency in brain structure segmentation.

Manual tracing measurements are commonly processed using approximate analytical formulas (Hofman, 1985; Ribeiro et al., 2013; Kazu et al., 2014; Herculano-Houzel et al., 2014; Maskeo et al., 2011; Manger et al., 2012), which have some drawbacks. The numerical approximations attempt to compute geometrical values, such as area and volume, from the areas and perimeters of contours in each slice. Unfortunately they cannot be used to perform analyses of detailed geometrical features, such as calculating the fractal dimension of the cortical surface (Wang et al., 2023).

Here, we introduce ’Stitcher’, a new deterministic surface reconstruction method and a pipeline to reconstruct 3D surfaces from manual tracing of contours in MRI brain slices. By combining automatic and manual methods, Stitcher is able to tackle cortices that are too complex and/or rare for fully automatic methods, and to generate much richer morphological information than what can be provided by analytical approximation formulas.

To demonstrate the capabilities of this tool, we chose to apply it initially to the brains of a group that pose a severe challenge to fully automated methods of surface reconstruction: cetaceans.

Cetacean brains are, in many ways, unique. Some species have the largest and most convoluted brains of the animal kingdom (Mortensen et al., 2014; Spocter et al., 2017). Additionally, neuromorphological patterns such as cortical thickness, white matter proportion, and sulci/gyri position vary greatly across species. Obtaining high-quality MRI data from cetaceans is logistically challenging and thus relatively rare. Collecting many individuals of the same species is even less likely. Therefore, there are no comprehensive imaging atlases or reference templates specifically for cetacean brains, and without those, automated algorithms would struggle to accurately segment brain structures.

Conversely, an accurate reconstruction of cetaceans’ cortical surfaces is indication that our tool is potentially applicable to the less convoluted brains of other non-model mammalian groups, such as carnivores and sirenians, as well as to widely-studied species, like humans and other primates.

To demonstrate usefulness beyond cortical reconstruction, we also show that Stitcher is capable of reconstructing other inner brain structures, such as the thalamus and ventricles.

This paper is structured as follows: In the next section, Materials and Methods, we introduce the Stitcher pipeline in detail. It is meant to be a self-contained description, linking to a code repository, that will enable any researcher to apply the pipeline to any suitable dataset.

This is followed by the results section, where the pipeline is validated against cetacean and non-cetacean species, and the resulting reconstruction is quantitatively compared to other methods. The section is also self-contained, insofar as the data presented there is new and of considerable interest to comparative neuroanatomical studies.

The relevance, limitations and future prospects of the Stitcher pipeline, and the significance of the cortical surface reconstructions done with it are detailed in the Discussion.

## Materials and Methods

### Data Acquisition and Processing

#### Specimens

The brains used in this paper were provided by the Brazilian Neurobiodiversity Network Initiative (https://neurobiodiversidade.org), recently established to remediate the lack of neuroanatomical descriptions in non-model animals. One brain was collected from each of three different species: Guiana dolphin (*Sotalia guianeneis*), Franciscana dolphin (*Pontoporia blainvillei*) and Steller sea lion (*Eumetopias jubatus*).

The two dolphins used in this study were collected with authorization from the Chico Mendes Institute for Biodiversity Conservation (ICMBio) and the Brazilian Institute of Environment and Renewable Natural Resources (IBAMA). The research was approved by the Committee on Ethical Animal Use of the Science Center of the Federal University of Rio de Janeiro (process number 01200.001568/2013-87).

The Steller sea lion was hunted as part of pest control at the coast of Shakotan (Hokkaido, Japan; Matsuda, 2021) in accordance with Japanese laws and regulations, a decision unrelated to the current study.

#### MRI Acquisition

For the two cetaceans, MRI images were generated in a 7 Tesla Scanner (Classic Magneton, Siemens Healthcare, Erlangen, USA) equipped with a 32-channel head coil (Nova Medical, Wilmington, USA) at the "Image Platform in the Autopsy Room" (PISA) facilities of the School of Medicine at the University of São Paulo.

For the Steller Sean Lion, brain scans were generated in a 3 Tesla Scanner (Magnetom Prisma, Siemens Health Care, Erlangen, Germany) with a 16-channel body coil at the facilities of the Medical School of Hokkaido University.

The MRI images were acquired by using the following protocol:

- Guiana dolphin: 3D-SPACE Repetition Time (TR) 2000 ms; Echo Time (TE) 109 ms; 0.38 mm isotropic spatial resolution; Field of View (FOV) 192x144; Bandwidth (BW) 610 Hz/px; Flip Angle (FA) 120; Acquisition Time (AT) 1 h 45 min (Avelino-de Souza et al., 2024);
- Franciscana dolphin: 3D-SPACE TR 2000 ms; TE 115 ms; 0.27 mm isotropic spatial resolution; FOV 102x102; BW 372 Hz/px; FA 120; AT 2 h 5 min;
- Steller sea lion: Axial T2 space TR 4000 ms; TE 410 ms; 0.78 mm isotropic spatial resolution; FOV 237x200; BW 700 Hz/px; FA 120; AT 12 min 24 s.

#### Processing

**Fig. 1:**
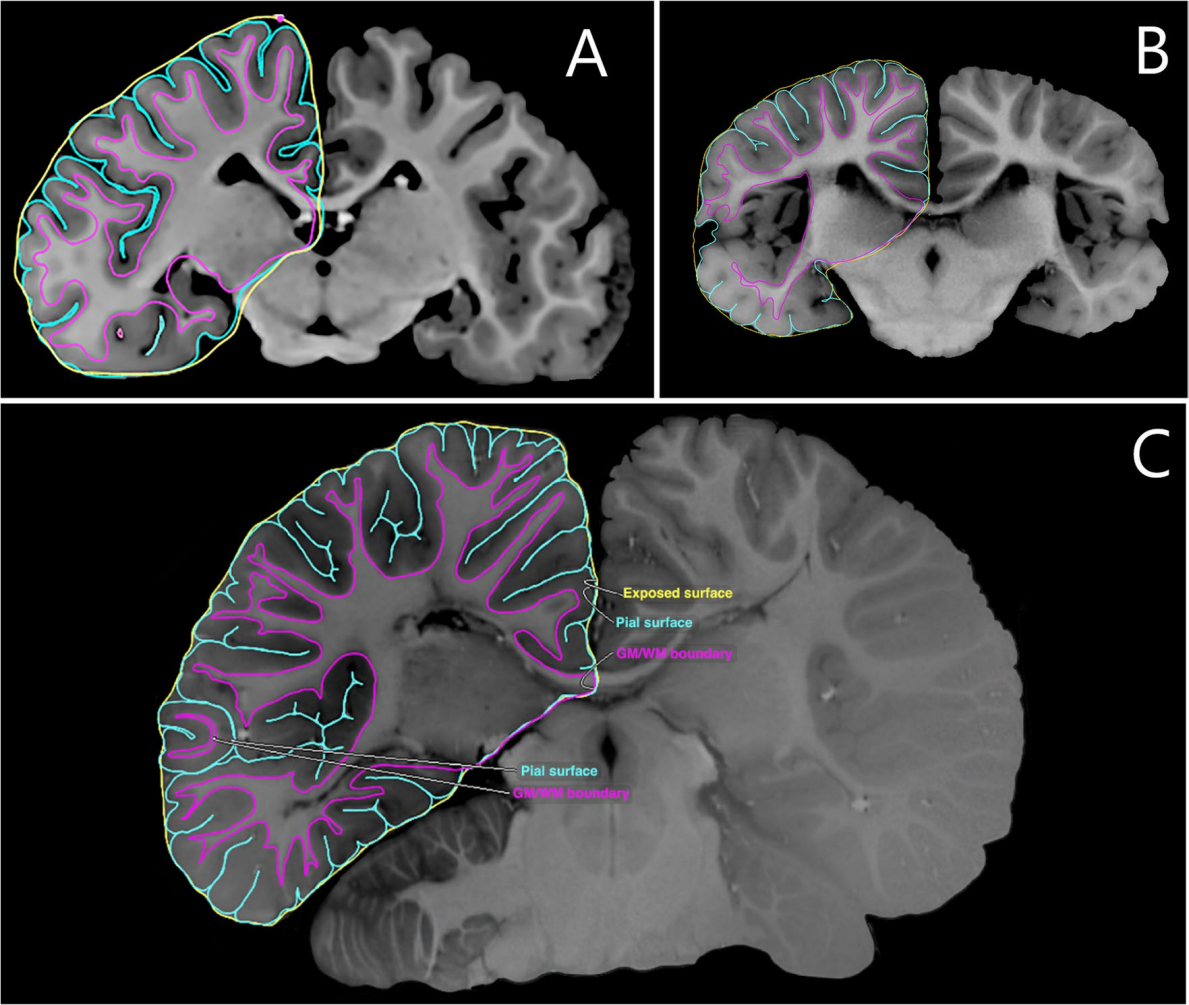
Examples of how the MRI and the manual segmentation of GM, WM and exposed surface ROIs look like for the three species in this study. **A** Steller sea lion. **B** Franciscana dolphin; adapted from Avelino-de-Souza (2023). **C** Guiana dolphin; adapted from Avelino-de Souza et al. (2024)

After image acquisition, images were used to obtain the contours of anatomically-defined regions of interest (ROI). This so-called segmentation consists of manually tracing the perimeters surrounding such areas (Fig. 1).

All brains were segmented by a single anatomist with the aid of a Wacom Cintiq Pro 13 (Wacom, Saitama, Japan) and the medical image software Osirix MD v.12.0.3 (Pixmeo, Geneva, Switzerland; Rosset et al., 2004). For *S. guianeneis*, the segmentation was performed every 5 slices, leaving a gap of 1.90 mm between consecutive segmentations. For *P. blainvillei*, the same spacing of 5 slices was used, leaving a gap of 1.33 mm. Lastly, *E. jubatus* had all its slices segmented, leaving a gap of 0.78 mm, the same value as the isovoxel length.

The manual segmentation data were then rearranged into two groups: full-resolution, when all available slices are used in the reconstruction, and low-resolution, when every other segmentation of the full-resolution is used. The low-resolution case is intended to mimic a scenario where data for cortical reconstruction is sparse.

**Fig. 2:**
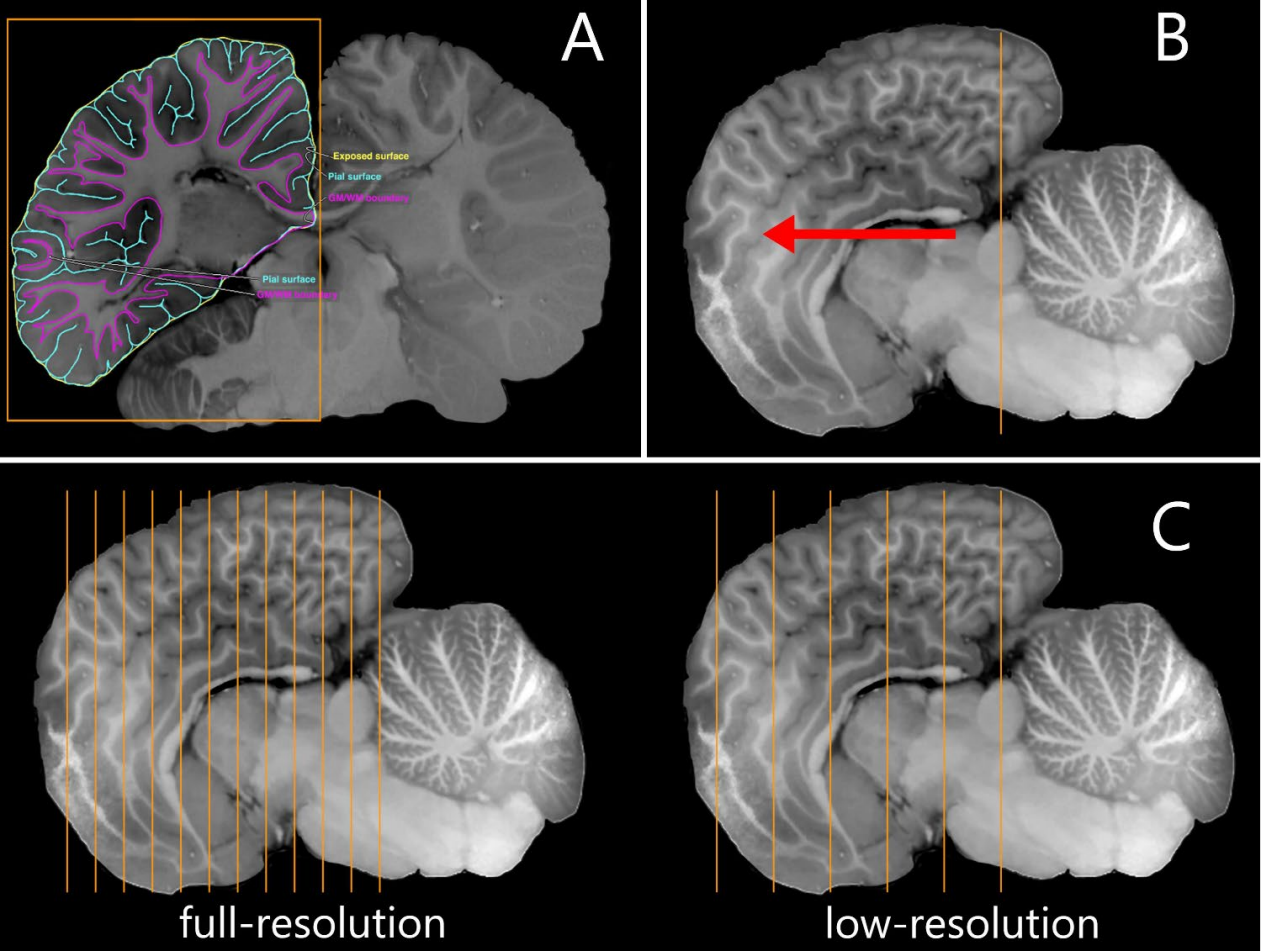
Visual guide of the data preparation for processing on Stitcher. **A** Example of a segmentation of gray matter, white matter and exposed surface ROIs; adapted from Avelino-de-Souza et al. (2024). **B** Sagittal view of the same brain; adapted from Avelino-de-Souza et al. (2024). The orange stripe shows the the first slice of segmentation and the red arrow indicates the direction of subsequent slices that must be segmented. **C** Stack of segmented slices, at full resolution (left), using all available slices; and at low resolution (right), using every other slice

Each individual segmentation was then saved as a JSON files, which were used as input in the reconstruction that followed. The full process is exemplified in Fig. 2.

### Stitcher

The Stitcher tool is an innovative software package for surface reconstructions, utilizing manual segmentation of contours in slice stacks, and generating a self-consistent lateral interpolating mesh surface as final output. This package (available at https://github.com/hmynssen/Stitcher) is structured as an open-source python library. Upon installation, it will provide 3 classes that can be employed together to 3D-reconstruct the brain. The core of the code implements a 2D search to find a minimum cost path that corresponds to a triangulation between contours in two consecutive slices (Fuchs et al., 1977), therefore creating a stitching pattern that connects all the vertices in both slices while preserving geometric and topological integrity. We use as the cost function the total edge length (therefore generating ’stretchy’ interpolation surfaces, analogous to cling film); but other functions, such as total surface area, can be easily implemented.

The minimum cost path might not be unique but all reconstructed surfaces corresponding to such absolute minimum path will be different triangulations of the same surface: think of a set of four points, two on each slice, forming a square being. Triangulation using either diagonal would be equally valid, but the final surface would not be exactly the same. In this sense, we can call Stitcher a deterministic reconstruction method for always creating equivalent reconstructions.

The first class, *Point* class, stores 3 number parameters to represent spatial coordinates for each vertex, and contains basic vector operations that simplify the code, such as vector addition, multiplication by scalar and dot product.

The next class is *Perimeter*, which contains a *numpy* (Harris et al., 2020) array storing a collection of *Points* corresponding to the contour exported by the segmentation tool. This sequence of *N* *Points* correspond to a closed polygon of $N-1$ edges and *N* vertices that encloses one of the three desired regions: GM, WM or exposed surface. From all the methods included in the *Perimeter*, three can be highlighted: Perimeter addition, that merges two polygons by connecting them in the shortest distance possible; plane self-avoiding correction, that removes edges that cross each other and creates the necessary new edges to preserve the polygon continuity; and orientation flip, that invert the order of the vertices and is used to guarantee that every polygon will be oriented clock-wise.

Lastly, the *Surface* class is centered around the method *build_surface* which performs the following algorithmic steps:

**Algorithm 1:**
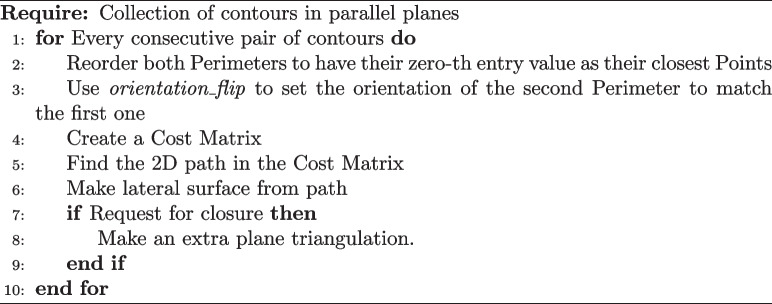
Class method *build_surface* logic

However, the minimum cost (5) may create self-intersections, which cannot exist in the real surface of a brain. For this reason, Stitcher imposes an extra condition in the search algorithm:

**Algorithm 2:**
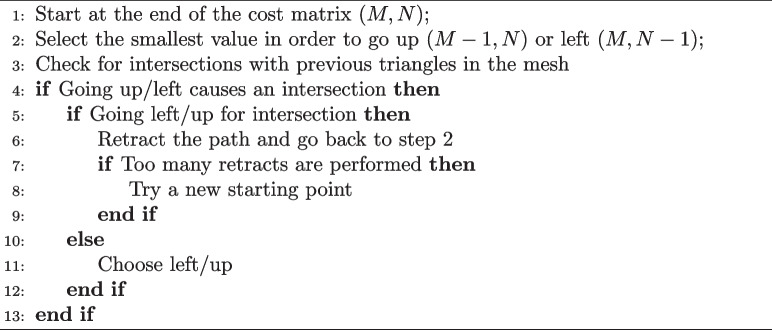
Self-avoidance logic

The retraction (6) is performed by removing the last entry from the path and setting it to have an infinite cost. If too many retractions are performed, as set by an end-user criteria, a new starting point is chosen. Usually, the starting point, *i.e.*, the first element of the Cost Matrix, is the closest distance between the above and below *Perimeters*. Due to the complexity of the brain shape, this starting point sometimes is not the best choice, leading to a mesh that would necessarily have a self-intersection. For that reason, a sorted list of of closest to furthest pairs of *Points* is computed and used in step 8 if necessary. The end-user may also provide the initial pair of points if so desired.

After step 5 from the first algorithm is done, step 8 is used to create a closed surface. Since the segmentation step only traces perimeter of a polygon, at least the final and last *Perimeters* must be tiled with a flat triangular mesh to prevent a hole from forming. Additionally, typically in the interior slices one or more splits/bifurcations of the segmentation contours will occur, two contours in one slice will need to be stitched self-consistently to a single contour in the next (much like trousers). Part of the single contour will need to be tiled with a triangular mesh to prevent a hole for forming.

#### Application of Stitcher for Subcortical and Non-Cortical Structures

**Fig. 3:**
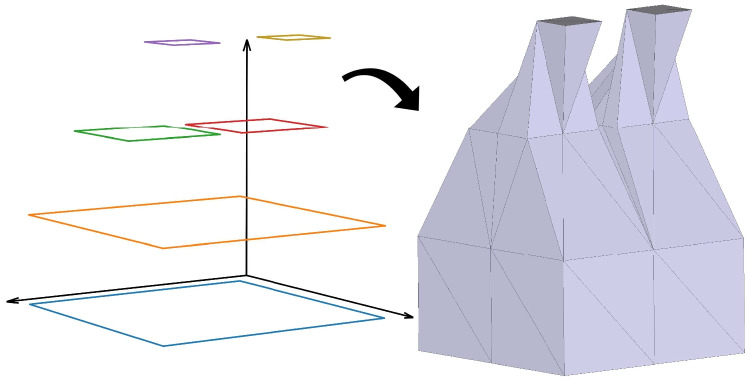
Example of a stack of arbitrary quadrilaterals (left side) and its reconstruction using Stitcher algorithm (right side)

Stitcher is capable of building any lateral surface given two polygons in parallel planes (Fig. 3). It can also reconstruct the WM surface, subcortical structures and non-cortical structures. As an example, we reconstruct the ventricle of the *Sotalia guianensis*, and calculate its area and volume. This multi-lobed but otherwise smooth structure poses no challenges to the algorithm.

Lastly, by using an atlas, an anatomist could identify structures of very low contrast, which are an even greater challenge for automatic segmentation tools. In this paper, superior and inferior colliculi, hippocampus, amygdala and thalamus from *Sotalia guianensis* were reconstructed to demonstrate this type of application.

#### Manual Corrections

**Fig. 4:**
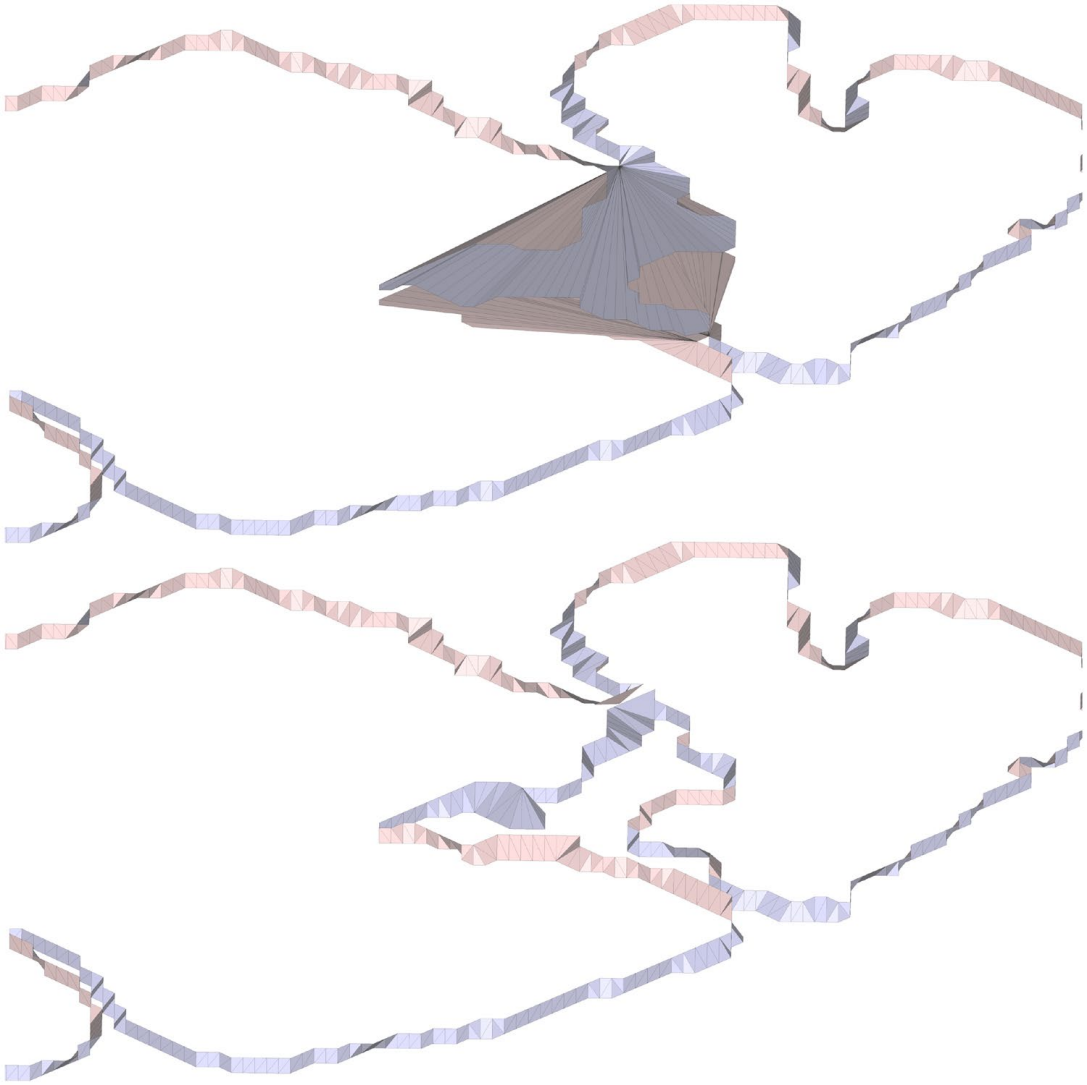
Knot artifact example: the final mesh (top) containing the artifact and the same mesh (bottom) after the manual correction of the input data

The Stitcher algorithm ultimately finds a self-avoiding mesh but that does not assure the absence of artifacts. In fact, one type of artifact can occur in highly gyrified brains that contain many perimeters/segmentation per MRI slice. Called knot artifacts, they can be easily identified visually as in Fig. 4.

**Fig. 5:**
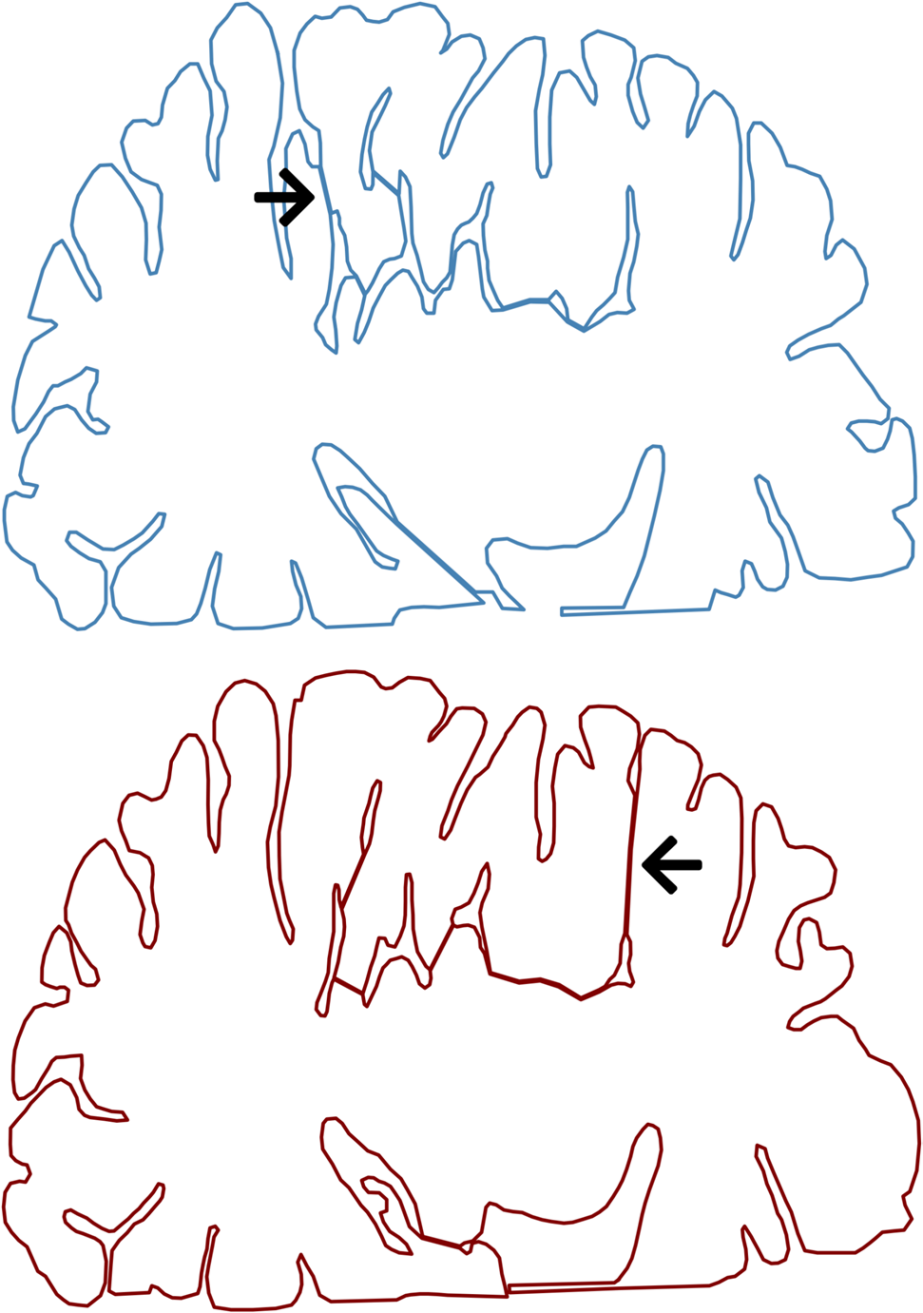
Example of inconsistent segmentation of a human subject gray matter. Two consecutive slices (top and bottom) have the beginning of a deep sulci at opposing sides (indicated by the arrows)

These artifacts increase the surface area and do not represent the real topology of the brain. However, the cause is not the stitching process itself, instead it is either a poorly performed segmentation or a failure in the algorithm of merging two *Perimeters* together, as in Fig. 5. The former can be fixed with a consistent segmentation of the gyri and sulci, while for the latter, new points must be inserted to create a bridge indicating where the connection between the two *Perimeters* should occur.

#### Post-Processing

The final reconstruction from Stitcher can be analyzed and manipulate in any mesh processing software. Here we used Meshlab (Cignoni et al., 2008) with the following pipeline to extract the geometrical value of volume and area of GM, WM and Exposed surfaces:

- Re-orient All Faces Coherently
- Close Holes
- Isotropic Remeshing
- HC Laplacian Smooth
- Taubin Smooth

The first two steps ensure that the surface is orientable and closed, which are necessary respectively for area and volume estimation. But the resulting raw surface will be at this point jagged in a ladder-like manner, which is clearly an artifact of the underlying voxelization. This will distort the area estimation, so for a more reliable result, one needs to smooth away the jagged edges. To that end, we first perform an isotropic remeshing (Hoppe et al., 1993) to re-tile the surface with more regularly-sized triangle edges; this does not alter areas or volumes but ensure that the ensuing smoothing, acting on the mesh vertices, happens homogeneously and isotropically over the entire surface. For the smoothing, we apply first a volume-preserving HC Laplacian flow (Vollmer et al., 1999) that diffusively evens out the positions of neighboring vertices; then a Taubin flow (Taubin, 1995), a modified Laplacian flow that evens out Gaussian curvature.

Visual inspection clearly indicates that the smoothed reconstructed surfaces are much closer to the original cortical surface than the raw ones, with their characteristic ladder-like pattern. The Smooth Surface remains closed and thus can have its geometrical values extracted again. Meshlab provides a python library, *pymeshlab* (Muntoni & Cignoni, 2021), making the above processing programmable via simple scripting. Here we used the graphical interface for visual inspection and *pymeshlab* for the all numerical data processing.

### Stitcher Usage

Stitcher relies only on sorting the stack of perimeters to start working. This can be done automatically by an adapted shadow casting algorithm that projects the *Perimeters* contained in one slice into the parallel slice below. All overlapping shadows should be grouped together to be reconstructed by Stitcher.

If this procedure is applied recursively through all pairs of segmentation slices, Stithcer will be able to reconstruct the ROI surface. Thus, Stitcher is able to produce a surface even if not all MRI slices of an acquisition sequence have been manually segmented. However having only a few segmented slices may reduce the anatomical information below the threshold necessary to properly reconstruct the structure. Thus, the number of slices that need to be traced must be determined by the anatomist and the end-user to balance the necessary detail with the required tracing workload.

This pipeline was implemented for the reconstruction of all cortical surfaces, *i.e.*, GM, WM and exposed surfaces, at both full- and low-resolution for comparison purposes. Also, the same pipeline was applied for Guiana dolphin’s hippocampus, amygdala, thalamus and ventricles.

### Analysis

#### Direct Measurements and Reference Values

The reconstruction results will be analyzed visually and numerically. For the GM surface, we extracted the area $A_t$ (total area) and the volume $V_t$ (total volume). For the other two, only one of the geometrical measures was needed. For WM, we only used the volume $V_{wm}$, and for the Exposed surface, the area $A_e$. The cortical thickness *T* was computed from GM and WM as:

1 $$\begin{aligned} \begin{aligned} T = \frac{V_t - V_{wm}}{A_t} . \end{aligned} \end{aligned}$$

Also, we compute the gyrification index (GI) as the ratio between $A_t$ and $A_e$.

In the absence of a full surface reconstruction of the brain regions of interest, it is possible to approximate (Ribeiro et al., 2013) their volume *V* and lateral surface area *A* using analytical formulas that take into account only the areas and perimeters of the contours in the slice stack:

2 $$\begin{aligned} \begin{aligned} A = \sum _{n}\{(S_{n+1}-S_n)^2 + [h(P_n+P_{n+1})/2]^2\}^{1/2} \end{aligned} \end{aligned}$$

3 $$\begin{aligned} \begin{aligned} V = \sum _{n} h[S_n+S_{n+1}+(S_n\times S_{n+1})^{1/2}]/3 \end{aligned} \end{aligned}$$

where $S_n$ and $P_n$ are the area and perimeter of a polygonal contour in slice *n* and *h* is the separation between adjacent slices. The Analytical Approximation (AA) process can thus be used in segmentation from consecutive MRI slices to approximate the value of $A_t$, $A_e$ and *T*,. This procedure has been applied to human cortices, and results were shown to be close to the corresponding real values (Ribeiro et al., 2013). As the gyrification increases, the cross-sectional images from MRI may display a series nested perimeters. Consider, for instance, an annular configuration in slice *n* of two nested contours, so that a region of (say) WM lies entirely inside GM; but within the WM region lies a smaller island of GM. This slice will then have two disjoint and closed contours of WM-GM boundary, each with an area and perimeter. In this case, the WM area $S_n$ and perimeter $P_n$ of the annular WM region to be used in Eqs. 2 and 3 must now read

4 $$\begin{aligned} \begin{aligned} S_n = S_{n1} - S_{n2} \end{aligned} \end{aligned}$$

5 $$\begin{aligned} \begin{aligned} P_n = P_{n1} + P_{n2}\text {,} \end{aligned} \end{aligned}$$

where the *n*_1_ and $n_2$ indices refer respectively to the outer and inner contour. More generally, if a slice includes multiple disjoint contours, their perimeters should always be added, and their areas summed algebraically with signs given by the parity of the degree of nesting.

This is a complication that usually does not arise often in the human and terrestrial mammal cortices we analyzed, but that clearly has to be taken into account for the more complex gyrification patterns often found in cetaceans.

After accounting for multiple contours, Eqs. 2 and 3 will compute the volume and area of any enclosed contour, with reasonable precision for actual cortical surfaces. But if we need a full mesh reconstruction of said surfaces, we need to apply the Stitcher or some equivalent method. In any case, we can directly compare the values of *V* and *A* obtained from the AA with those derived from the Stitcher reconstruction.

#### Effects of Different Resolutions

By varying the gap between slices, we also vary the amount of information provided to reconstruct the brain. On one extreme, by having only the first and last slices, the mesh would yield a reconstruction resembling a truncated cone. On the other extreme, we would have mesh whose triangles would be so tiny we could consider it a continuous and smooth surface.

The minimum gap value to get proper cortex lies between these extremes in this spectrum of resolutions. To approximate it, we’ll use the condition:

6 $$\begin{aligned} \begin{aligned} \text {gap}_{\textit{min}} \simeq T \end{aligned} \end{aligned}$$

The rationale behind this are spatial frequency of the image and practical aspects of measurements. Firstly, for any given brain MRI, we expect for structures come in and out from the screen at a certain rate as the slices are looked through. This is the spatial frequency of structures and for the folded GM with thickness *T*, a sulci will be defined/identified with $2 \times T$ (to account for GM walls on both sides). In order to obey the Nyquist-Shannon Theorem (Shannon, 1949), we take half of $2 \times T$, which is just *T*, to properly represent the image signal.

Lastly, it is important to have an initial guess on the gap before starting the segmentation. Therefore, by making the gap equals the thickness, one may simply use a ruler on any image processing software and directly measure the gap estimation.

## Results

**Fig. 6:**
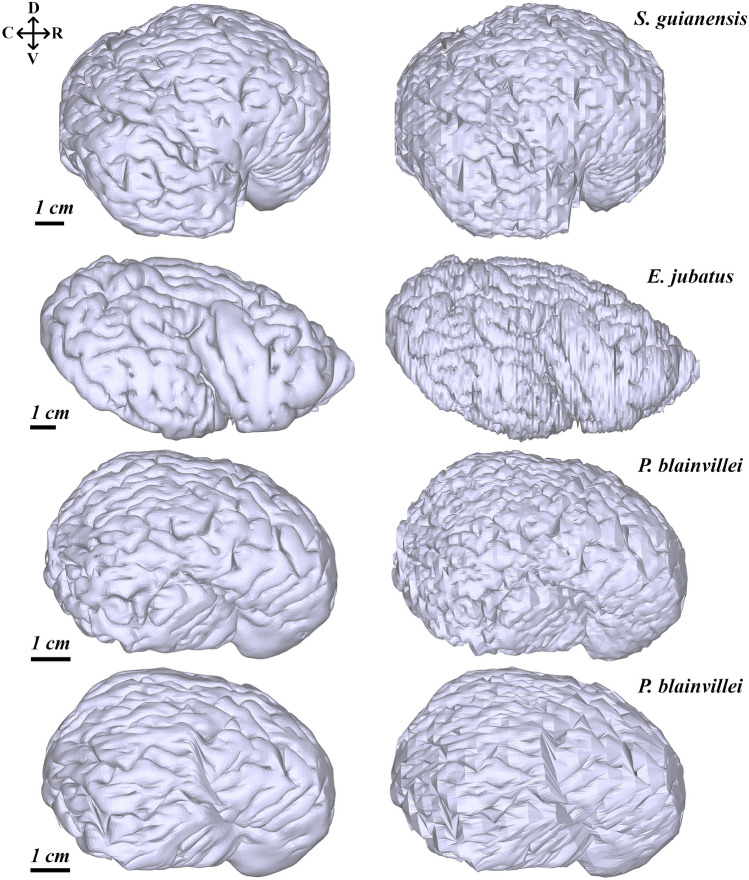
Lateral view of pial surface reconstructions by Stitcher for: *S. guianensis* on the first row; *E. jubatus* on the second row; and *P. blainvillei* on the third (full-resolution) and forth rows (low-resolution). The left column are the smooth surfaces and the right column prior to smoothing (Raw). Orientation abbreviations: caudal (C), rostral (R), dorsal (D), ventral (V)

The main result of this paper, the reconstructed cortical surfaces of Steller sea lion, Guiana dolphin and Franciscana dolphin, are shown in Fig. 6. None of these hemisphere reconstructions present any significant artifacts when visually inspected. However, the low-resolution Franciscana reconstruction showed some artifacts, low gyrification, and a distinct stretch pattern in contrast to its full-resolution version.

The values for the geometric parameters^1^ describing the reconstructed cortices of the three species analyzed are presented in Table 1. In all cases we include the values for the both the raw and smoothed reconstructed cortices. The values calculated using the analytical approximation (’AA’) are also included for reference. Note that there is very little variance in the volumes across all three methods, while for areas and thicknesses the smoothed values are close to the analytical approximation, but much higher (for areas) and lower (for thicknesses) than the raw values. This is due to terraced appearance of the latter, a consequence of the initial voxelization, which artificially inflates areas by making the raw reconstructed surface more irregular.

**Table 1:** Summarizing of the numerical results: total volume $V_t$, total area$A_t$, exposed area $A_e$, avarage thickness *T* and gyrification index GI

	**Species**			
	***S. guianensis***	***P. blainvillei***^a^	***P. blainvillei***^b^	***E. jubatus***
Raw $V_t\ (\text {mm}^3)$	248839.64	75437.32	75319.80	220978.90
Smooth $V_t\ (\text {mm}^3)$	244544.52	74252.04	74242.30	218558.29
AA $V_t\ (\text {mm}^3)$	250930.97^c^	75843.67	76333.91	221304.23
Raw $A_t\ (\text {mm}^2)$	83647.02	32378.35	29370.42	55710.77
Smooth $A_t\ (\text {mm}^2)$	65945.84	25919.50	24758.39	44269.64
AA $A_t\ (\text {mm}^2)$	63801.96^c^	24652.15	24491.72	41534.63
Raw $A_e\ (\text {mm}^2)$	24251.47	11274.83	10876.45	27872.76
Smooth $A_e\ (\text {mm}^2)$	23100.20	10796.45	10672.01	23906.51
AA $A_e\ (\text {mm}^2)$	22864.80^c^	10111.26	10046.42	22851.35
Raw $T\ (\text {mm})$	1.73	1.45	1.59	2.27
Smooth $T\ (\text {mm})$	2.10	1.75	1.83	2.79
AA $T\ (\text {mm})$	2.26^c^	1.92	1.96	3.06
Raw GI	3.45	2.87	2.70	2.03
Smooth GI	2.85	2.40	2.32	1.85
AA GI	2.79^c^	2.44	2.44	1.82

^a^Full-resolution *P. blainvillei* using every segmentation slice at spacing 1.3 mm

^b^Low-resolution *P. blainvillei* using every other segmentation slice at spacing 2.7 mm

^c^Data from (Avelino-de Souza et al. 2024)

**Fig. 7:**
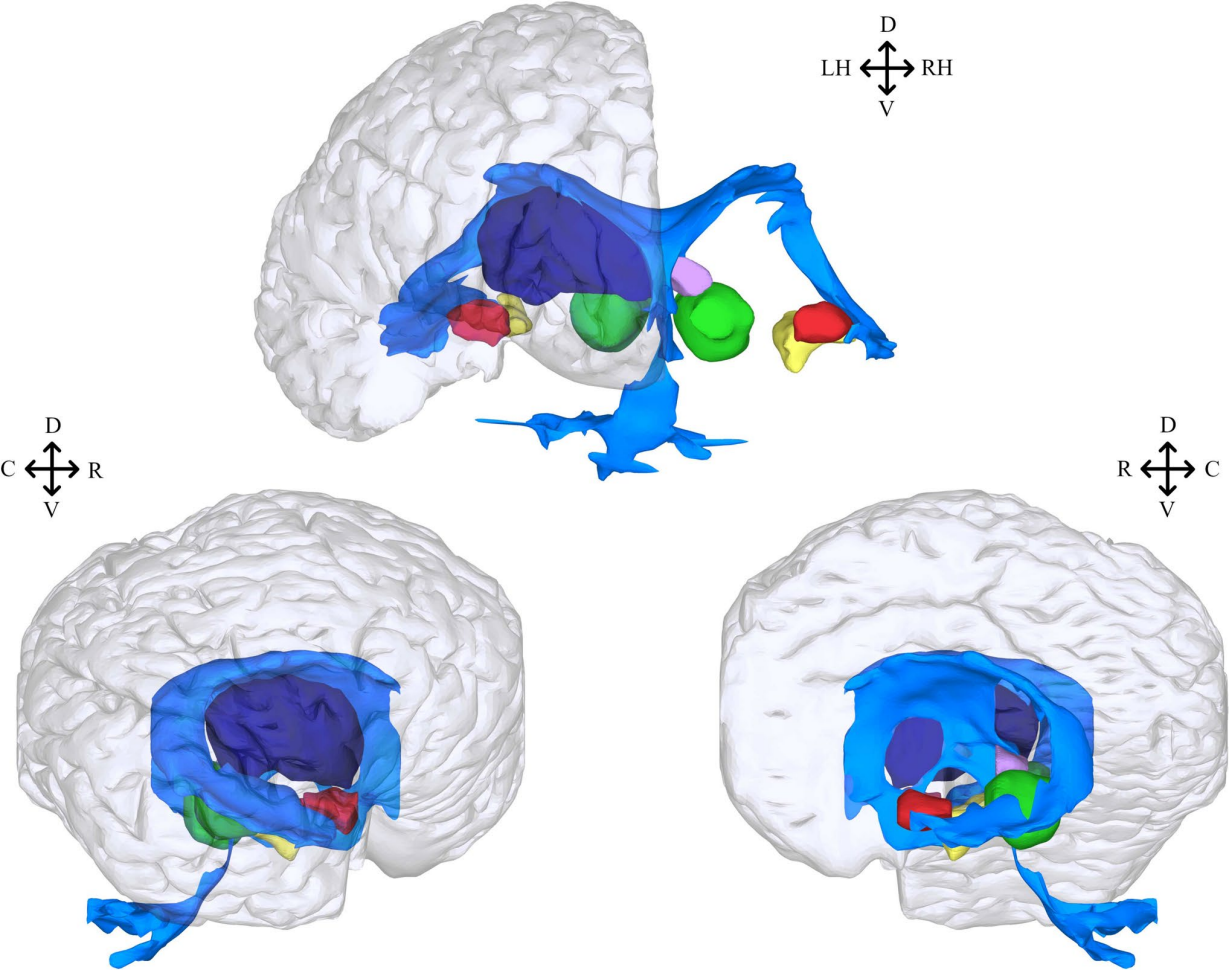
Stitcher 3D reconstruction of *S. guianensis*’s subcortical and non-cortical structures. Structures can bee seen in frontal (top), lateral (bottom left) and sagittal (bottom right) views with a translucent overlay of the pial surface. All structures have been colored to facilitate identification: ventricle (color blue), superior colliculus (color violet), inferior colliculus (color green), hippocampus (color yellow), thalamus (color dark blue) and amygdala (color red). Except for the ventricle and thalamus, structures from both hemispheres were included to allow proper visualization and avoid overlapping, specially in lateral and sagittal views. Orientation abbreviations: caudal (C), rostral (R), dorsal (D), ventral (V), left hemisphere (LH), right hemisphere (RH)

Stitcher was also successfully applied to reconstruct subcortical structures, such as the hippocampus, amygdala, superior and inferior colliculi and thalamus of *S. guianensis*. The hippocampus and amygdala are located at temporal lobe level as part of the limbic system. Additionally, both colliculi integrate the mesencephalon, and the two thalami are part of the diencephalon. Additionally, the often neglected ventricular system of the same species was reconstructed, as shown in Fig. 7, being a bounded by a continuous and closed surface. Similarly to what was reported for other dolphins, reconstruction of the ventricular system reveals that the structure is short, but wide in the level of the temporal lobe (Cozzi et al., 2017). The lateral ventricles are circular and the third ventricle appears as a ring shaped structure due to the interthalamic adhesion connecting the two thalami. The third ventricle connects with the forth ventricle thought the cerebral aqueduct, a tubular structure that expands dorsally and laterally, creating a triangle-like shape in the lateral view. The fourth ventricle then joins the central canal of the spinal cord (see Fig. 7 for a sagittal view).

**Table 2:** Summarizing of subcortical and non-cortical structures numerical results: meshes volumes and areas for *S. guianensis* subcortical and non-cortical structures

	***S. guianensis***	
	**Area (cm** $^{\varvec{2}}$**)**	**Volume (cm** $^{\varvec{3}}$**)**
Ventricle	84.93	6.18
Superior Colliculi^a^	3.80	0.33
Inferior Colliculi^a^	13.10	2.96
Hippocampus^a^	9.20	0.74
Thalamus	23.60	7.72
Amygdala^a^	7.28	0.94

^a^Areas and volumes for these structures refer to the summation in both hemispheres

All these structures were grouped in a single image (Fig. 7). Except for the ventricles, with its characteristic structure of connected projecting horns, they all have relatively simple rounded shapes, and fit satisfyingly together like pieces of a puzzle. This strongly suggests that the reconstruction process generated no artifacts. Indeed, the relative anatomical positions of the subcortical structures are largely preserved, resembling the generalized mammalian *bauplan*. Numerical results of volumes and areas for all structures are summarized in Table 2.

## Discussion

We have presented Stitcher, a novel tool that can process 2D manually segmented brain data and use it to generate the corresponding 3D surfaces, even for the most morphologically complex brains, such as those found in some cetaceans and pinnipeds. Since this paper presents results relevant for both computational methods, and cortical and subcortical neuroanatomy, we have divided the discussion into three sub-sections to properly address each subject.

### Cortical Results

#### Reconstruction Validation and Limitations

We applied Stitcher to reconstruct the cortical surfaces of three species, totaling three cortices in full-resolution and an extra reconstruction for one specimen in low-resolution. For the former, the resulting meshes exhibited a remarkable resemblance to real brains, without any significant visible artifacts.

In contrast, when applying Stitcher for low-resolution *P. blainvillei* cortical reconstruction, we found some issues: knot artifacts, decreased gyrification, and the erasure of smaller structures visible in both in the full-resolution version and in the photographs of the cortex. This is not unexpected, and reflects a loss of anatomical information from the region between the slices. Skipping too many MRI slices forces Stitcher to interpolate between slices that are too dissimilar. Stitcher creates surfaces formed by planar triangles that can be stretched, translated and rotated, but cannot be bent. This means that the reconstruction is faithful only when the gap between slices is smaller than or equal to the radius of the typical smallest gyrus in the cortex.

In this manner, there is a trade-off between manual labor and quality of results. Accurate cortical surface reconstruction require numerous and closely spaced traced slices, and this requirement becomes more stringent the thinner and more gyrified a cortex is. This is, probably, the most significant practical limitation of our method, as the necessary tracing can become very time-consuming, especially for bigger brains, as the case of large cetaceans. In any case, for a precise anatomical description this step remains necessary, unless the user purposely wants to remove small structures and/or is only looking for certain numerical aspects (see more in “Quantitative Anatomical Description” section).

Another necessary aspect of the manual tracing is the accurate identification of the ROIs. In our work, we utilize MRI with good enough contrast and resolution so that we were able to properly trace the contours. In future works, it may be necessary to adjust the MRI parameters according to the MRI machine and biological material one possesses to avoid incorrect segmentation. Stitcher does not fix the segmentation and mistakes on this step of the process will reflect in possible artifacts on the final mesh.

#### Quantitative Anatomical Description

Our numerical results can be split into two groups: comparison across reconstruction methods (Raw, Smooth and AA) and comparison between full- and low-resolution reconstructions.

Firstly, by comparing methods, we observed that volume and exposed area remained nearly invariant regardless the methods. This happens because these two metrics are less affected by precise delimitation of the borders on the segmentation step.

However, for total area $A_t$ and, consequently, the cortical thickness *T*, the precise delineating of the boundary between regions is a critical factor. In the case of the Raw reconstruction, it inherits to some extent the jagged edges from the underlying voxels. These cubic spikes are clearly artifacts of the reconstruction pipeline, and (as can be clearly seen by visual inspection) are not present in the actual physical cortical surface, which are usually smooth at these voxel-size length scales. Smoothing the Raw results erases, or at least reduces, these artifacts.

Numerically, the smoothing brings $A_e$, $A_t$ and *T* measurements for Stitcher-reconstructed surfaces closer to those obtained from the AA method. This is unsurprising, since smoothing makes adjacent slices more alike, which is similar to the fundamental assumption used to derive AA. This highlights another important point for the usage of smoothing filters: it makes AA and reconstructions methods compatible, which, in turn, makes comparison with results from the literature that use AA methods less susceptible to methodological incompatibility.

In the second comparison group (full- and low-resolution), the summary of geometric parameters vary relatively little with a small tendency for reduction on estimates based on surface reconstruction. Possibly, the reduction of slices progressively removes small structures while preserving the main cortical components, thus largely conserving the numerical results. To support this, we compare both gaps of 1.33 mm (full-resolution) and 2.66 mm (low-resolution) with the cortical thickness *T* of around 1.80 mm for the Smooth *P. blainvillei*. In the first case, the gap is smaller than *T* meaning that the proposed condition (check “Effects of Different Resolutions” section) was satisfied, while for the second case, the opposite holds true.

### Non-Cortical and Subcortical Structures

We have also applied Stitcher to the reconstruction of non-cortical structures, which presents their own set of challenges. For example, direct descriptions of the ventricular system is often impractical in fresh brain examinations, due to its interior, multi-lobed and liquid-filled nature. Consequently, ventricular system reconstructions in cetaceans are scarce and date from the 60s, using vinylite or latex injections to produce a cast mold of the ventricles (McFarland et al., 1969). These methodologies can distort the ventricular shape, through material shrinkage, gravity deformation of drained ventricles, and resin leakage (McFarland et al., 1969). They also cause damage to surrounding structures during mold extraction.

Here, by using Stitcher, we were able to reconstruct the ventricular system of *Sotalia guianensis* from MRI scans. The non-invasive nature of our methodology enables the analysis of the structure without causing damage or altering the morphological properties of the ventricular cavities and neighboring brain regions.

Similarly, delineating subcortical structures, namely the superior and inferior colliculi, hippocampus, thalamus and amygdala from MRI scans alone presents a demanding task. Creating meshes of these structures aids in visualizing them in 3D, which can help anatomists mitigate tracing errors, thereby enhancing the accuracy of neuroanatomical measurements. To corroborate this, (Avelino-de Souza et al., 2024) found equivalent results in terms of volume for these structures using analytical calculations.

Additionally, 3D reconstructions are valuable tools to allow a comprehensive understanding of the organization and connections of brain structures within the brain. From a comparative perspective, by comparing 3D reconstructions of brains from different species, we can identify similarities and differences in brain organization and connectivity, shedding light on evolutionary relationships and adaptations across species.

### Stitcher as an Alternative Reconstruction Tool

The Stitcher pipeline was applied to new, rare and highly complex cortical MRI datasets, and produced unprecedented reconstructions for cetaceans and pinnipeds cortices. To produce such results, it relied on manually segmented contours, which can be drawn for any structure by an expert. Since the contours are its only input, Stitcher is a generalist tool, capable of reconstructing not only cerebral cortices, but also other 3D structures, as long as contours are available. Some examples are shown here, but in principle it could be applied to other brain structures, to non-mammalian brains, or even inanimate objects.

However, manual segmentation is known to have some limitations: Although it is more accurate and reliable than current automated methods, manual tracing require expertise in neuroanatomy, can be time-consuming, prone to bias, and impractical for large datasets (Keller & Roberts, 2010). In any case, manual segmentation is a necessary first step even for automated tools, as they need to be trained and adjusted. This emphasizes the potential usage of Stitcher in sparse imagining data, and as potentially the only tool capable of generating complex surface reconstructions for a broad class of anatomical studies.

#### Stitcher vs Generic Reconstruction Tools

In some scenarios, the ROIs will not contain structures smaller than the voxel size. In this case, it is possible that an interpolation between manually segmented ROIs will produce an accurate result for the surfaces between ROIs. Software such as 3D (Slicer Fedorov et al., 2012), ITK-SNAP (Yushkevich et al., 2006) and Mango (Lancaster et al., 2011) could be used to perform this task.

Also, the same software provide reconstruction mechanism that can be directly applied to those ROIs, which are implementations or adaptations of the Marching Cubes (Lorensen & Cline, 1987) or the Flying Edges (Schroeder et al., 2015). Additionally, for very uniform ROIs in terms of pixel brightness, manual segmentation might not be needed, and threshold segmentation could be a much faster alternative.

However, if the interpolation fails or if the ROIs contains structures with sub-voxel width, these generic tools will fail to produce accurate surfaces. This is specifically true in the case of the GM, where the gap between opposite sulcal walls may fit inside a voxel. In these cases, the surfaces produced will not include the sulci to its full depth.

#### Stitcher vs Specialized Reconstruction Tools

In contrast to the generic approach, some specific methods targeting mainly the GM surface have been developed. However, the two main alternative reconstruction tools, FS (Fischl, 2012) and FMRIB Software Library (FSL; Jenkinson et al., 2012), are heavily calibrated towards human data-sets, preventing their use in our data in their current form. Nevertheless, FS and FSl are robust and well-known tools, amply documented, and it is possible that they could be adapted for other species with proper adjustments and training. Recently, FSL and FS has been combined into a single semi-automatic pipeline, *precon_all* (https://github.com/neurabenn/precon_all), to attempt cortical reconstructions in non-humans.

However, published adaptations so far seem to still be limited to cortices less gyrified then cetaceans and pinnipeds (Roumazeilles et al., 2022). Thus, currently, FS and FSL-derived methods are not able to process all the diversity of brain morphologies present in nature. Nonetheless, they can still be used to statistically validate Stitcher. As a follow-up study, we will conduct a comparison with standard tools and human datasets (Mynssen, 2023), to benchmark Stitcher’s accuracy.

A fundamental way in which Stitcher differs from other tools based on probabilistic and/or minimization approaches is that it generates deterministic results: the same input will always generate the same output. This means that Stitcher could also be less biased in quantifying uncharted data. This situates it as uniquely useful to investigate new species with unusual cortical morphologies, understudied brain structures or even neuropathologies.

## Conclusions

Due to its flexible implementation, Stitcher is a new, viable and innovative reconstruction pipeline for cortical and non-cortical structures of non-model species. Focusing specifically on cetacean brains, Sticther proved itself effective for processing manually segmented data, providing reliable reconstructions in situations where automatic tools may fall short.

In the spirit of open science, we provide the Stitcher pipeline as an open-access (MIT Licensed) python library, allowing a variety of specialists to access a new domain of analysis for non-model species. Cortical and subcortical reconstructions could be a new leading resource in comparative neuroanatomy in the near future.

## Information Sharing Statement

The data used in this study was partially described in a previous study (Avelino-de-Souza, 2024). Stitcher code is freely available at https://github.com/hmynssen/Stitcher.

## Data Availability

The Stitcher source code implemented in python can be found at https://github.com/hmynssen/Stitcher. The repository include instructions for installation and usage.

## Footnotes

1 Volume $V_t$, GM area $A_t$, exposed area $A_e$, cortical thickness *T* and gyrification index $\text {GI} = \frac{A_t}{A_e}$, see Mota and Herculano-Houzel (2015)
